# *TERT* Genotype Polymorphism: A Glance of Change Egyptian MDS Outcomes

**DOI:** 10.31557/APJCP.2021.22.5.1547

**Published:** 2021-05

**Authors:** Nadia El Menshawy, Shaimaa El-Ashwah, Mohamed A. Ebrahim, Metwaly Ibrahem Mortada, Ahmed Ramez, Doaa M. Atia

**Affiliations:** 1 *Department of Clinical Pathology, Hematology Unit, Faculty of Medicine, Mansoura University, Egypt. *; 2 *Clinical Hematology Unit, Department of Internal Medicine, Faculty of Medicine, Mansoura University, Egypt. *; 3 *Medical Oncology Unit, Department of Internal Medicine, Faculty of Medicine, Mansoura University, Egypt.*

**Keywords:** TERT, SNVs, myelodysplatic syndromes

## Abstract

**Background:**

Myelodysplastic Syndromes (MDS)are clonal hematologic disorders characterized by genetic instability and ineffective hematopoiesis associated with telomere dysfunction. We aimed at investigating the association between the rs2242652 single nucleotide variant of the *TERT* gene and susceptibility for MDS, as well as its prognostic impact and relation to disease phenotype.

**Methods:**

Genotyping analysis was carried on 100 MDS patients recruited at Mansoura Oncology center, in addition to 100 healthy subjects for detection of rs2242652 variant of *TERT* gene on chromosome 5 by real time PCR following the protocol of Custom TaqMan^®^ SNP Genotyping.

**Results:**

The rs2242652 *TERT* genetic polymorphism was associated with an increased risk of MDS (odds ratios 2.6 for genotype GA, 6.4 for genotype AA). The majority of AA homozygous mutant variant were associated pancytopenia (88%), poor risk cytogenetics (92%) and High/very high IPSS-R score (88%). At the end of follow-up (median 30 months), 14% of the cases transformed to secondary AML. The rate of leukemic transformation was significantly associated with the mutant AA genotype (93% of transformed cases, 52% of AA genotype cases; P< 0.0001). Survival outcome was inferior in AA mutant genotype (median 14 months, 95% CI: 12-16 months) to the GA genotype (median 30 months, 95% CI: 26-33 months) and those of the GG genotype (median not reached), P<0.001.

**Conclusion:**

Our study shows an intriguing and previously unrecognized association between rs2242652 TERT mutation and MDS risk. The presence of rs2242652 mutation defines a subgroup of patients with aggressive disease phenotype and dismal outcome. Further research is recommended to elucidate underlying pathologic mechanisms and to define an efficient therapeutic target.

## Introduction

MDS represent hematological clonal disorder that typically affect elderly. They arise as a consequence of genetic mutations (more frequently chromosome alterations) in a pluripotent hematopoietic stem cell. Clinical manifestations of MDS are influenced by cytopenia-related complications and by progression into AML (Sperling et al., 2017).

Telomeres are a repetitive hexanucleotide (TTAGGG) sequences which bind many specialized proteins to the ends of the chromosome (Yang et al., 2012). Telomeres prevent coding sequence erosion and protect chromosomal stability by aiding in complete chromosome replication and regulating gene expression (Stewart et al., 2012).

Telomerase is an RNA-dependent DNA polymerase that synthesizes telomeres. The telomerase complex, consists of an RNA template (TERC), and an enzymatic subunit (telomerase reverse transcriptase, *TERT*) (Jafri et al., 2016). Reduced level of TERC is sufﬁcient to cause telomere diseases, such as dyskeratosis congenita, aplastic anemia, and idiopathic pulmonary ﬁbrosis while up regulation of *TERT* expression have a critical role in tumor formation and chemotherapy outcome (Townsley et al., 2014). Enhanced telomerase activity has been reported in the majority of cancers and is associated with immortalization, and resistance to apoptosis through elongation of telomeres (Jafri et al., 2016).

Accumulating evidence proved that altered telomere plays a crucial role in bone marrow failures, leukemias and MDS (Lansdorp, 2017; Menshawy et al., 2020). Telomere length is affected by the presence of single nucleotide variants (SNVs) in the *TERT* gene influencing activity and/ or expression (Mosrati et al., 2015a; Ozturk et al., 2017).

It has been suggested that telomerase activity and *TERT* expression may have a role in pathogenesis of MDS and impact the prognosis of MDS patients (Vasko et al., 2017). However, the SNV (rs 2242652) in *TERT* gene expression was not explored in MDS. Our study adopted a genotype-based approach, with the objective of determining the contribution of rs2242652 allele of *TERT* gene to MDS risk in Egyptian population. We aimed also to address its impact on prognosis of MDS as well as its association with disease characteristics and leucocyte telomere lengths.

## Materials and Methods

*Patients and Methods*

*Patients*

This study was carried on 100 MDS patients (54 males, 46 females), recruited at oncology Mansoura university center from April 2015-untill Mars 2018, in addition to 100 age, sex-matched healthy subjects as reference control. Diagnosis of MDS was established according to 2008 WHO diagnostic criteria (Vardiman et al., 2009). Informed consents from fall participants ’guardians in study were obtained and approved by Mansoura medical ethics Committee (MMEC) of the faculty of medicine. Follow up was at least 2 years to assess prognosis and outcome. 

*Methods*

*Sampling*

DNA was extracted from peripheral leucocyte using Thermo scientific Gene *JET* Whole Blood Genomic DNA Purification kit according to the protocol of manufacturer’s instructions. The extracted DNA was preserved frozen at - 20 C. quantification of DNA samples by Nano-Drop instrument, 1000 spectrophotometer (Thermo Fisher Scientific Inc., Wilmington, NC, USA).

*Genotyping analysis real time -PCR*

DNA extracted with specific SYBR^®^ Green master mix was used for detection of rs2242652 variant of *TERT* gene on chromosome 5 was followed the protocol of Custom TaqMan^®^ SNP Genotyping Assays under guide of manufacturing kits. For assurance of quality control purpose, the order of amplified DNAs sample was randomized on plate with duplication of 5 samples all over the runs to satisfy our result finding. PCR plates were acquired on DNA-Technology DT Prime4 real time instrument, software v7.6.

*Leucocyte telomere length assay*

Measurement of relative telomere length by Real time quantitative PCR following the technique by Cawthon et al., (2002).

Relative T/S values were calculated according to 2^-ΔΔ Ct^

• ΔCt= Ct (caliprator) – Ct (unkown sample).

• ΔΔCt = ΔCt (telomere) – ΔCt (single copy gene).

*Cytogenetic analysis*

Interface fluorescence in situ hybridization (FISH) (del(5q)/ –5, del(7q)/ –7, trisomy 8, del(20q), trisomy 1/1q+) were performed according to the manufacturer’s instructions on mononuclear cells of BM aspirates. and Conventional cytogenetic by G-banding, depend on culture and harvest methodology with trypsin and Geimsa stain. The different Probes purchased form (Vysis, London, UK), analysis of at least 100 metaphases for every case by an expert and professional highly specialized staff at international Canadian accredited lab. Cell images were captured using a CCD camera (Photometrics SenSys camera) using CytoVision system for image analysis (Applied Imaging).

*Statistical analysis*

Data were analyzed running IBM-SPSS© for windows version 19.0. A two-sided p value of <0.05 was required for statistical significance. The Chi Square Test was used for testing the relation between categorical variables. Mann–Whitney U test or Kruskal–Wallis H test were used for comparison between two or more groups. Survival was determined by the Kaplan-Meier test, the Log Rank test was used for comparison. Independent hazards of different prognostic factors were tested by the Cox’s regression model.

## Results

The study was conducted prospectively on 100 cases with MDS in addition to 100 control subjects. The mean age of studied cases was 56±11 years, including 54% males & 46% females. Control subjects were matched with the cases for age (mean 58±9 years; p=0.2) and sex (males 55%, females 45%, p=0.9). The baseline characteristics of studied cases are shown in table 1

**Figure 1:**
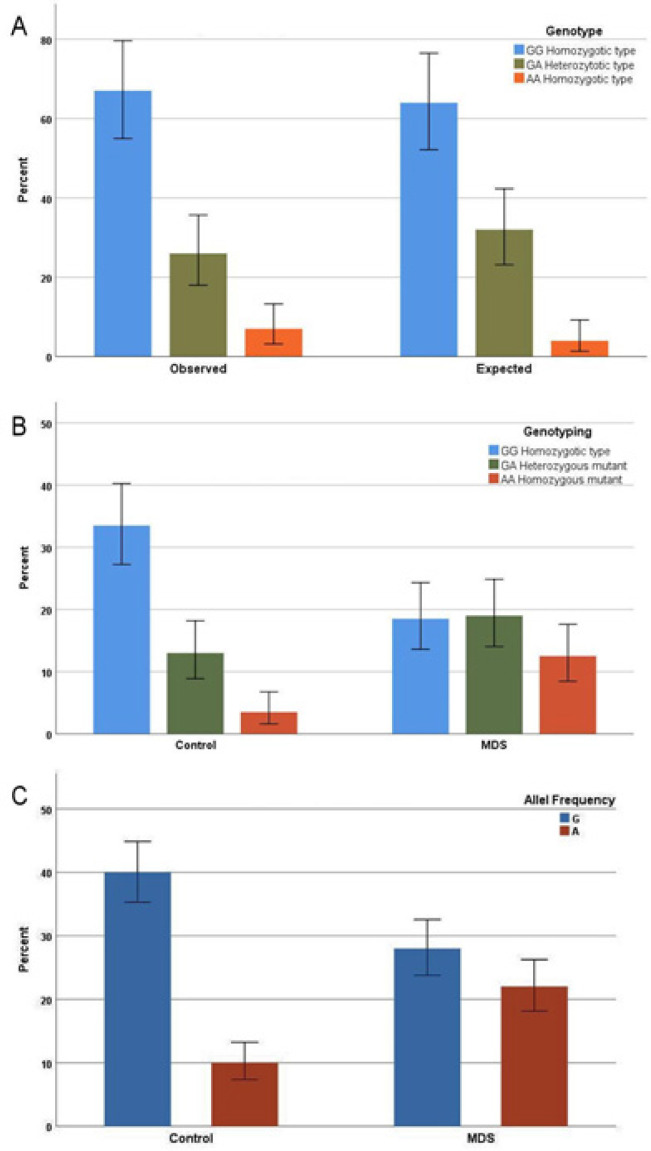
(A), The distribution of genotypes in control subjects inn relation to Hardy Weingberg Equilibrium (p=0.1); (B), The distribution of genotypes in MDS cases versus control subjects (Odds ratio for GA genotype 2.6, 95% CI 1.4-5; Odds ratio for AA genotype 6.4, 95% CI: 2.6-16); (C), The frequency of mutant allel A in MDS cases and control subject (Odds ratio for Allel A 1.7, 95% CI: 1.4-2)

The genotype distribution of *TERT* polymorphism in control group did not deviate from Hardy-Weinberg equilibrium (P=0.1). Analysis of the differences in frequency distributions of genotypes and alleles between cases and controls showed that *TERT* mutations were associated with a significantly increased risk of MDS; Odds ratios were significant for both the genotype distribution (2.6 for genotype GA, 6.4 for genotype AA) and for the allele distribution (1.7 for A) as shown in Figure 1.

**Figure 2:**
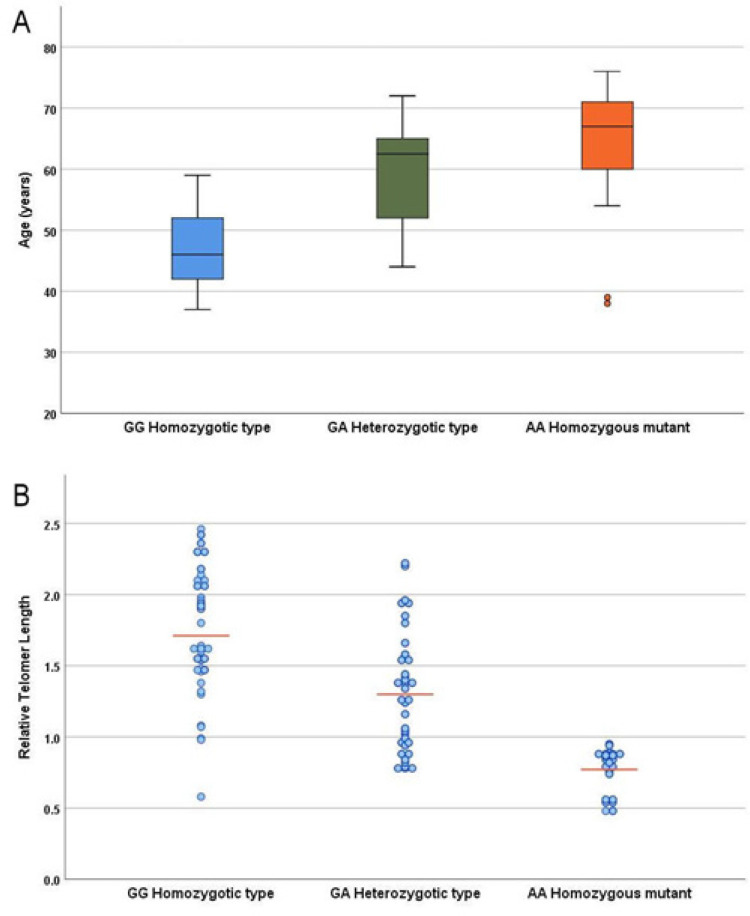
Relation of Genotypes to Age (p< 0.0001; post hoc: GG vs. GA, p=0.001; GG vs. AA, p=0.001; GA vs. AA, p=0.1) (A) and relative telomer length (p< 0.0001; post hoc: GG vs. GA, p=0.01; GG vs. AA, p=0.0001; GA vs. AA, p=0.001) (B) in MDS cases

The mutant homozygous genotype (AA) and the heterozygotic genotype (GA) were significantly associated with older age (mean 64±10 and 60±8 years respectively) versus GG genotype (mean 47±6 years, P=0.001) as shown in (Figure 2A). A significantly shorter telomere length was found in the genotypes with mutant allele (Figure 2B).

**Figure 3:**
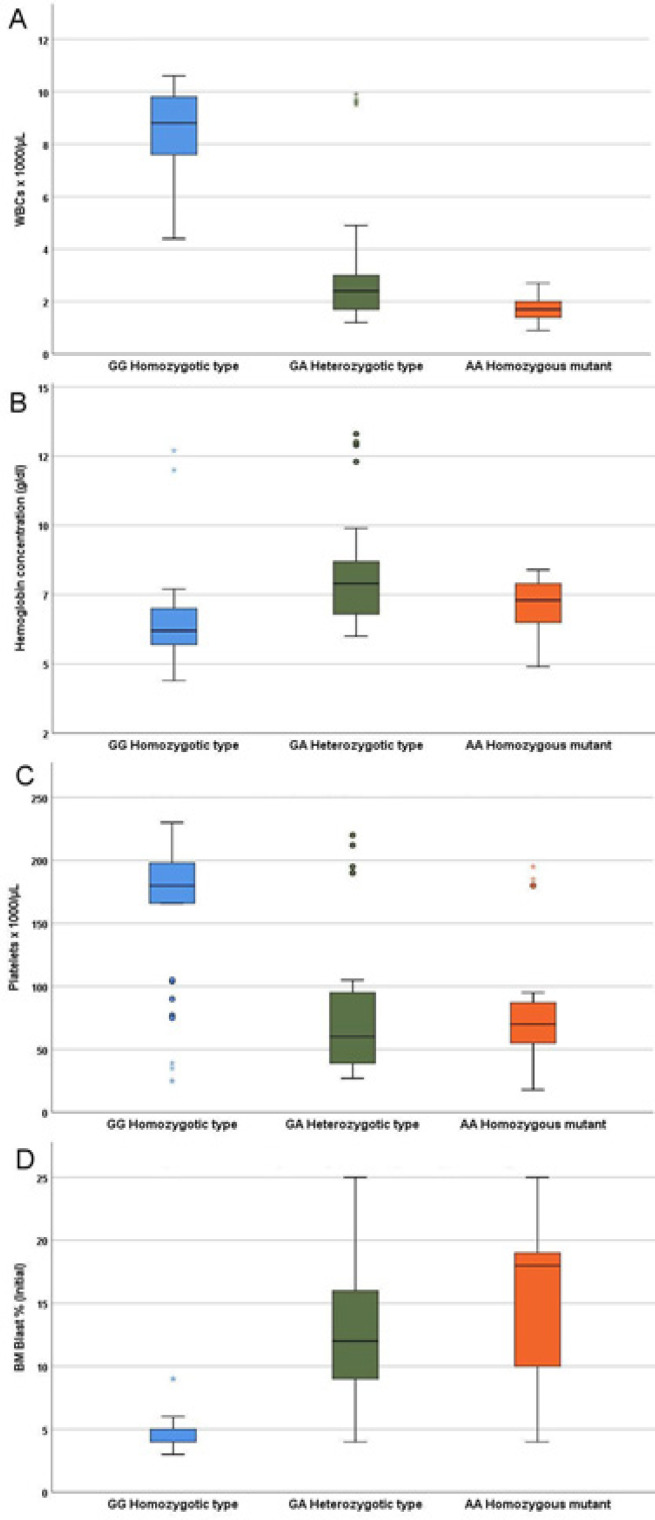
Relation of Genotype to Peripheral Blood Count and Bone Marrow Blast Cells Percentage: WBC count (p= 0.0001; post hoc: GG vs. GA, p=0.001; GG vs. AA, p=0.001; GA vs. AA, p=0.006); Hemoglobin concentration (p= 0.0001; post hoc: GG vs. GA, p=0.001; GG vs. AA, p=0.5; GA vs. AA, p=0.03); Platelets count (p= 0.0001; post hoc: GG vs. GA, p=0.001; GG vs. AA, p=0.001; GA vs. AA, p=1.0); Bone marrow blasts (p< 0.0001; post hoc: GG vs. GA, p=0.001; GG vs. AA, p=0.001; GA vs. AA, p=0.01)

**Figure 4:**
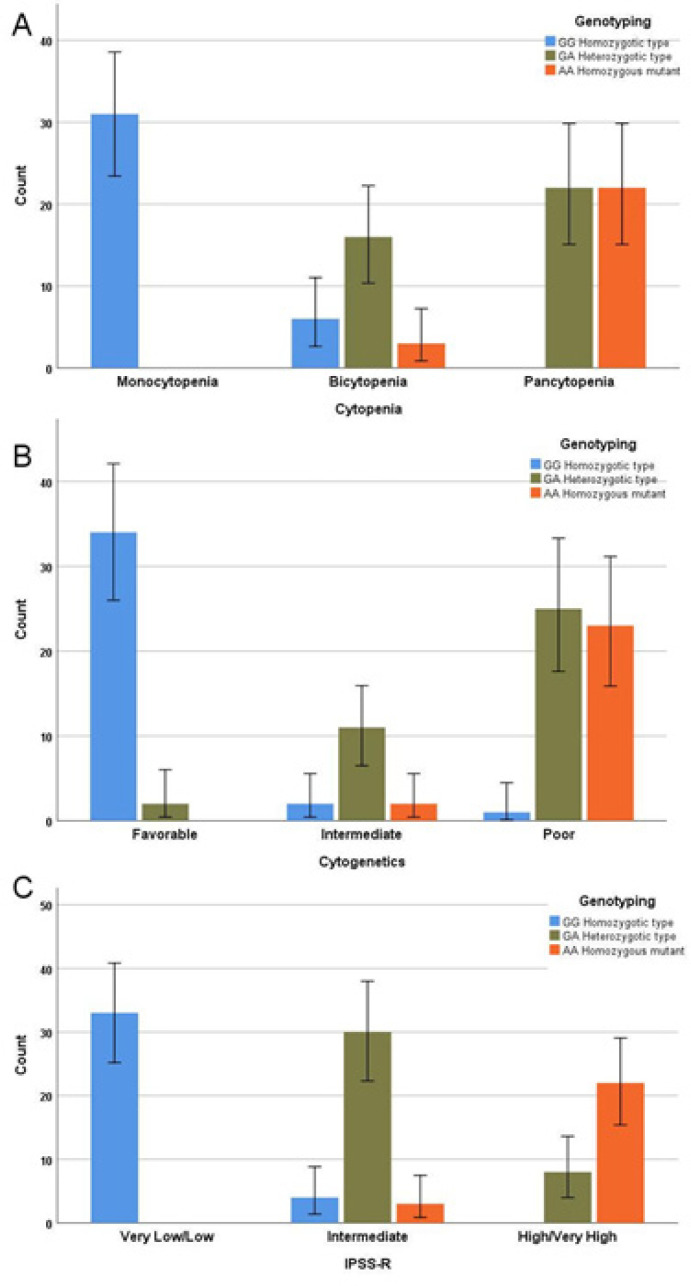
Relation of Genotypes to Cytopenia (p<0.0001), Cytogenetics (p<0.0001) and Revised-International Prognostic Scoring System (p<0.0001) in MDS Cases

Regarding the disease related parameters, *TERT* SNV rs 2242652 AA was associated a significant reduction in mean hemoglobin concentration, lower WBCs and platelets count, pancytopenia (88%), poor risk cytogenetics (92%), MDS with excess blasts (88%), MDS with multilineage dysplasia (12%), and high /very high IPSS-R score (Figures 3, 4).

**Figure 5:**
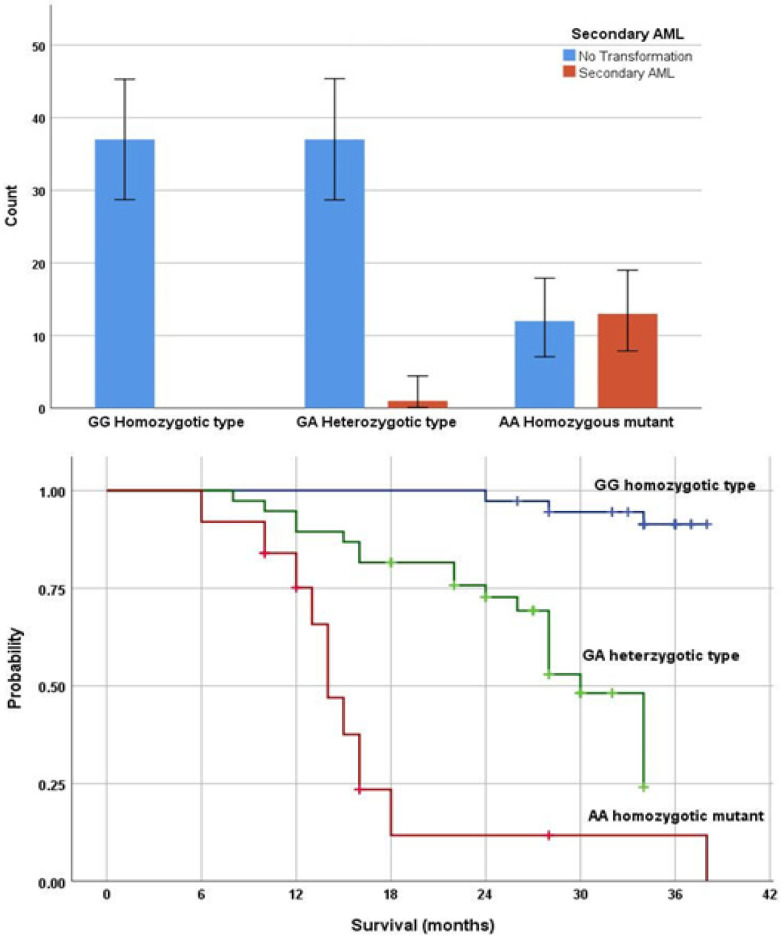
Relation of Genotypes to Transformation to Secondary Leukemia (p<0.0001) (A) and Overall Survival (p<0.0001) (B).

At the end of follow-up (median 30 months), 14 cases (14%) transformed to secondary AML. The rate of leukemic transformation was significantly associated with the mutant AA genotype (93% of transformed cases, 52% of AA genotype cases; P< 0.0001). The median overall survival of studied cases was 38 months (95% CI: 32-44 months). The survival outcome was inferior in AA mutant genotype (median 14 months, 95% CI: 12-16 months) to the GA genotype (median 30 months, 95% CI: 26-33 months) and those of the GG genotype (median not reached), log rank =60, P<0.001 (Figure 5). Univariate survival Cox regression analysis identified 6 variables that significantly predicted short overall survival including older age, bi/pancytopenia, intermediate/poor cytogenetics, bone marrow blast cells >5%, intermediate/high/very high IPSS-R and mutant genotype distribution (HR 9, 95% CI 2.4-26.5 for heterozygous genotype GA; HR 23, 95% CI 11.7-36.4 for mutant genotype AA). However, in multivariate analysis genotype distribution was not independently associated with an impact on overall survival (Table 2).

**Table 1:** Baseline Characteristics of Studied MDS Cases

Total = 100 cases		No	%
Sex	Male	54	54%
	Female	46	46%
Diabetes		24	24.00%
Hypertension		31	31.00%
Smoking		20	20%
Cytopenia	Monocytopenia	31	31%
	Bicytopenia	25	25%
	Pancytopenia	44	44%
WHO classification	MDS-SLD	15	15%
	MDS-RS	7	7%
	MDS-MLD	22	22%
	MDS-EB-1	13	13%
	MDS-EB-2	37	37%
	MDS del(5q)	6	6%
Cytogenetics	Favorable	36	36%
	Intermediate	15	15%
	Poor	49	49%
IPSS-R	Very Low/Low	33	33%
	Intermediate	37	37%
	High/Very High	30	30%
Transformation to Secondary AML	No Transformation	86	86%
	Secondary AML	14	14%
Mortality	Died	40	40%
Genotyping	GG Homozygotic type	37	37%
	GA Heterozygotic type	38	38%
	AA Homozygous mutant	25	25%

MDS-SLD, myelodysplastic syndrome-single lineage dysplasia; RS, ringed sideroblastic; MLD, multilineage dysplasia; MDS-EB, myelodysplastic syndrome Excess blast

**Table 2:** Hematological Characterization of MDS Patients

	Mean ± SD	Range
Age (years)	56 (11)	37-76
WBCs x 1,000/µL	4.9 (3.5)	0.9-10.6
Hb conc. (g/dl)	6.8 (1.7)	3.9-12.8
Platelets x 1,000/µL	108 (68)	18-230
BM Blast % (Initial)	10 (6)	3-25
Relative Telomer Length	1.3 (0.5)	0.48-2.46

**Table 3:** Univariate and Multivariate Analysis of Prognostic Factors of Overall Survival

	Univariate			Multivariate		
	HR	95.0% CI	P	HR	95.0% CI	P
Age group (≥ 65 years)	5.2	2.7-10.2	< 0.001			
Cytopenia			0.01			
Bicytopenia	1.6	1.2-8.9	0.03			
Pancytopenia	1.9	1.2-11.1	0.02			
BM blast percentage >5%	8	2.2-19.3	0.1			
Cytogenetics			< 0.001			
Intermediate	3.01	0.51-17.7	0.16			
Poor	31.5	1.9-22.9	0.001			
IPSS-R			< 0.001			<0.001
IPSS-R (intermediate)	10.1	2.1-48.2	0.003	10.1	2.1-48.2	0.003
IPSS-R (high/very high)	49.1	24.6-64.2	< 0.001	49.1	24.6-64.2	<0.001
Genotyping			< 0.001			
Heterozygous (GA)	9	2.4-26.5	< 0.001			
Homozygotic mutant (AA)	28	11.7-36.4	< 0.001			

## Discussion

The activation of telomerase is a vital step during cellular immortalization and malignant transformation in human cells, and many human malignancies are characterized by elevated *TERT* expression (Young, 2010; Ye et al., 2017). The *TERT* gene sequence in general is thought to be indicative of an individual’s susceptibility to cancer, and epidemiological studies have identified associations between specific *TERT* polymorphisms and cancer development (Yin et al., 2012). It has been reported that the rs2242652 allele of *TERT* influences telomere length, which has in turn been linked to a number of diseases including cancers (Heidenreich and Kumar, 2017; Wu et al., 2017a; Yang et al., 2019a; Roggisch et al., 2020).

Earlier studies have reported that the rs2242652 *TERT* genetic polymorphisms were associated with increased risk of non-hematological malignancies including: melanoma (Nan et al. 2011), estrogen receptor negative breast cancer (Bojesen et al., 2013), prostatic carcinoma (Hazelett et al., 2014), glioma (Mosrati et al., 2015a), lung cancer (Ye et al., 2017), esophageal cancer (Wu et al., 2017b), urothelial carcinoma (Roggisch et al., 2020), and thyroid carcinoma (Yang et al., 2019b).

Among the hematopoietic malignancies, MDS is most likely to be associated with mutations of telomere maintenance genes (Alter et al., 2010). Therefore, the rs2242652 *TERT* genetic polymorphisms is postulated to be particularly relevant to MDS, however, to date, there no report on such association.

In this study, we investigated the association between the rs2242652 SNP variant of the *TERT* gene and the susceptibility for MDS and its relations to clinicopathologic features of the disease. We found that the rs2242652 *TERT* genetic polymorphism was associated with an increased risk of MDS in an Egyptian patient population (odds ratios 2.6 for genotype GA, 6.4 for genotype AA and 1.7 for the allele distribution A). Association of rs2242652 SNP with MDS risk may in part be explained by influencing the telomere function resulting in chromosomal instability. When genomic instability ensues, the vast majority of cells undergo apoptosis, although in occasions cell may survive and become tumorigenic (Stewart et al., 2012). In addition, beyond immortalization, *TERT* also possess telomere independent functions in tumor formation, regulating Wnt-dependent transcription, mitochondrial function, apoptosis, and DNA damage response. It was also shown that *TERT* interacts with NFκB and co-activates the expression of several genes that are critical for cancer progression (Mosrati et al., 2015b; Ozturk et al., 2017).

A significant interaction was found between genotypes and age in MDS cases, the mean age was significantly older in AA homozygous mutant genotype (mean 64±10) followed by the GA heterozygotic type (mean 60±8) and the younger age group was found in the GG homozygous type (47±6), p=0.001 A concordant significantly shorter telomere length was found in the genotypes with older age (mutant allele).

When the data were analyzed with respect to disease related parameters, the mutant genotype was associated with a significant reduction in WBCs and platelets counts, and significant elevation in bone marrow blast cells. The majority of AA homozygous mutant variant were associated pancytopenia (88%), poor risk cytogenetics (92%) and High/very high IPSS-R score (88%). Collectively these data indicate that this genotype represents a high-risk abnormality, linked to aggressive clinicopathologic features of MDS. This association has not been previously explored in MDS meanwhile, in solid tumors *TERT* mutations were associated with significant poor clinical parameters which predict poor prognosis and may represent a novel therapeutic target (Shimoi et al., 2018).

At the end of follow-up (median 30 months), 14 cases (14%) transformed to secondary AML. The rate of leukemic transformation was significantly associated with the mutant AA genotype (93% of transformed cases, 52% of AA genotype cases; P< 0.0001). The biological mechanism that could account for this striking high rate of transformation and clinical aggressiveness is the development of rapidly acquired genetic changes that may promote progression to AML as consequences of genomic instability.

The overall survival was inferior in AA mutant genotype (median 14 months, 95% CI: 12-16 months) to the GA genotype (median 30 months, 95% CI: 26-33 months) and those of the GG genotype (median not reached), P<0.001. However, in multivariate analysis genotype distribution did not add prognostic information to the IPSS-R score after adjusting for age, cytopenia, cytogenetics, and bone marrow blast cells. This suppression of the prognostic value of the mutant genotype could be explained by the strong association with poor IPSS-R score.

Some limitations of the current study have to be taken into consideration when interpreting the results. First, the sample size was relatively small. Second the relation of this molecular marker to other genetic and epigenetic prognostic markers that have been defined in MDS was not examined. Lastly, the predictive value of this molecular marker was not evaluated in relation to different treatment modalities.

In conclusion, our study shows an intriguing and previously unrecognized association between rs2242652 *TERT* mutation and MDS risk. The presence of this mutation defines a subgroup of patients with aggressive phenotype, strikingly high frequency of transformation to AML and a short survival. Finally, the MDS-associated molecular marker identified here might be useful as a prognostic biomarker and potential therapeutic target warranting future studies.

## Author Contribution Statement

Nadia El Menshawy: design the study, organize team work, revision of manuscript., Shaimaa El-Ashwah, Ahmed Ramez :collect clinical data of patients and follow up. Mohamed A. Ebrahim: do statistical of study and revision of manuscript, Metwally Ibrahem Mortada and Doaa Attia wrote the manuscript.

## Participants included in the study

Informed consent was obtained from all patients
